# Time to Redefine Organic Agriculture: Can’t GM Crops Be Certified as Organics?

**DOI:** 10.3389/fpls.2018.00423

**Published:** 2018-04-10

**Authors:** Amjad M. Husaini, Muhammad Sohail

**Affiliations:** ^1^Genome Engineering Lab, Division of Plant Biotechnology, Sher-e-Kashmir University of Agricultural Sciences and Technology of Kashmir, Shalimar, India; ^2^Department of Biochemistry, St Hilda’s College, University of Oxford, Oxford, United Kingdom

**Keywords:** transgenic plants, recombinant DNA technology, genetically modified organisms, sustainable agriculture, environmental pollution, climate change

## Abstract

The challenges of sustainable food production without damaging the environment for a growing human population have increased considerably. The current agricultural practices involving chemical fertilizers and even organic farming are not sustainable in the long run and can have deleterious effects on the environment. Thus, new, innovative solutions need to be identified and propagated for tackling this. Among such innovations, that can complement conventional as well as organic farming methods, are genetic modification (GM) and aquaculture. Yet, GM technologies often face resistance from civil groups owing to an ‘unknown’ fear, akin to Frankenstein’s monster. How real is this fear? Our discussion rests on basic questions like, why can’t ‘organics’ include GM crops that do not require chemical inputs for cultivation, and can GM crops like Golden rice qualify to be ‘organic’ if cultivated through organic practices? Do we need to rethink organic agriculture in the context of the present and future challenges of 21st century?

## Introduction

Industrial revolution, forward-looking land planning and new political strategies have played a key role in helping meet global food demands of the rapidly-increasing human population. Global population was less than 1 billion in 1800; the increasing food demand over the next century was met by increasing the area of land under cultivation. In 1900, the world population increased to 1.6 billion; consequently, the following century witnessed increase in crop productivity through “green revolution,” involving extensive farm-mechanization and the use of high-yielding fertilizer-responsive varieties. In 2017, the global population touched 7.5 billion and is estimated to increase to 9.5 billion by 2050. Not only that, the available agricultural land is likely to reduce with time and we may be compelled to use relatively less hospitable lands to meet our agricultural needs, exerting further pressure on the need for developing alternative environment-friendly, sustainable and politically-acceptable farming strategies for food production. Despite the backing of a large section of the scientific community, the idea of GM organics has failed to set roots in the mainstream agricultural practices, and, therefore, a sustained campaign in the form of providing further supporting arguments and evidence is needed for its wider acceptance. These arguments form the basis of the discussion below.

## Can Gm Crops Offer Traits That Mitigate Adverse Effects of Climate Change? Can These Also Facilitate in the Adoption of Resource Conservation Technologies?

The short answer is yes. However, it is important to develop an unfathomable understanding of the available technologies and use them in designing climate-resilient crops (reviewed in Husaini et al., 2010, 2011). The vital traits for plants to adapt to climate change include heat, drought, water-logging and salinity tolerance, water- and nitrogen-use efficiency, early vigor, cold and frost tolerance, pest and disease resistance (Husaini, 2014).

Genetic modification (GM) techniques provide access to a greatly increased and diverse gene pool for developing plant varieties with desired traits. Examples are numerous: submergence-tolerant rice with *SUB 1A* gene (Xu et al., 2006; Dar et al., 2013) can produce good yields even after a fortnight under water, conditions that would destroy most other types of rice; GM maize “SmartStax^TM^,” produced by engineering eight genes, with insect pests and imparting herbicide tolerance has already received approval from the United States Environmental Protection Agency (EPA) and regulatory authorization from the Canadian Food Inspection Agency (CFIA) in 2010; GM maize, DroughtGard^TM^ (Stein and Rodríguez-Cerezo, 2010), first planted in the United States in 2013, has increased 15-fold from 50,000 hectares in 2013 to 810,000 hectares in 2015, reflecting its increasing acceptability by the farming community; Water Efficient Maize for Africa (WEMA), led by the African Agriculture Technology Foundation (AATF), is developing new African drought-tolerant maize varieties with the best GM technology available and will share these with African countries (James, 2016); GM rice and canola that fix nitrogen more efficiently need less nitrogen fertilizer, thus reducing N_2_O emissions, which has an almost 300 times higher potential to cause global warming than CO_2_ and stays in the atmosphere for more than 100 years (Nicholas, 2006; Reay et al., 2012).

Genetic modification technology can also help develop plant varieties with better adaptability to farm operations like ‘no tillage’ or ‘reduced tillage,’ practices that are helpful in growing crops under water-limited environments. Genetically-modified Roundup Ready^TM^ herbicide-resistant soybean crop accounts for nearly 95% of no-till area in Argentina and United States. With consumption of each liter of tractor diesel, 2.75 kg of CO_2_ would otherwise have got added into the atmosphere. Therefore, besides the fuel savings on account of fewer tractorization runs, relative to conventional crops, it results in reduced CO_2_ emissions (Brookes and Barfoot, 2017). Furthermore, crops engineered for enhanced disease tolerance, pest resistance, and better nitrogen fixation ability, could reduce the input of chemical pesticides and fertilizers, thus ‘tending toward’ organic farming.

A lower requirement for fertilizer also means lower input costs and greater profit for farmers; engineering cereal crops that could be self-supported by biological nitrogen fixation is a way forward (Geddes et al., 2015). A global meta-analysis of 147 studies for the last two decades has shown that, on average, GM technology adoption has increased crop yields and farmer profits by 22 and 68% respectively, while reducing use of chemical pesticides by 37% (Klumper and Qaim, 2014).

## Is It Time to Redefine Organic Agriculture?

Organic agriculture is a strongly consumer-driven sector. The well-being and common motive of producing organic food is largely associated with the absence of chemical residues and its presumed higher nutritional value (Hughner et al., 2007). It uses techniques first used centuries back, such as crop rotations, composted animal manures and green manure crops, in ways that are economically sustainable only in some parts of today’s world (Gold, 2016). Thus, the focus on health appears to be the most common motive for organic food across different regions of the world (Sirieix et al., 2011). If this be the case, then can GM crops such as Golden rice qualify to be ‘organic’ if cultivated organically?

Are the principles behind organic farming uniform across the world and can these be altered with time?, especially in view of the challenges posed by the growing human population and climate change, as well as updated scientific data. According to International Federation of Organic Agricultural Movements [IFOAM] (2005), “Organic Agriculture is a production system that sustains the health of *soils, ecosystems* and *people.*” It usually prohibits the use of compounds that are produced by chemical synthesis, such as antibiotics, growth hormones, genetically modified organisms, synthetic pesticides and fertilizers (Martin, 2009; Treadwell et al., 2015). However, in some cases the use of copper(II) sulfate, boric acid, pyrethrin, lime sulfur, sodium percarbonate, rotenone (highly toxic to aquatic life and possibly linked to Parkinson’s disease), bromomethane and azadirachtin (affects beneficial insects) (Seaman and Sideman, 2013; Ruddock, 2016) is approved. Thus, on the one hand, the use of these chemicals is permitted as exceptions against the fundamental notion of organic farming, while on the other, in its 12th Scientific Conference, IFOAM (1998) had issued the Mar del Plata Declaration to exclude the use of GMOs in food production and agriculture. The argument behind this declaration was that “it involves unacceptable threats to human health, negative and irreversible environmental impacts, the release of organisms of an un-recallable nature, removal of the right of choice, both for farmers and consumers, violation of the farmers’ fundamental property rights and endangerment of their economic independence – practices which are incompatible with the principles of sustainable agriculture as defined by International Federation of Organic Agricultural Movements (IFOAM)”^1^.

Based on the above discussion, this appears to be an appropriate juncture to revisit this nearly two decade old IFOAM declaration in light of the more recent findings, while also dispelling the fears about GM crops, as supported by several eminent scientific bodies and more than 100 Nobel Laureates. The international reference standards of IFOAM and Codex Alimentarius act as minimum guidelines for organic farming; they are not set in stone, and can be complemented by additional, national or private standards, which may differ from region to region, or even country to country. For example, EU regulations have some exceptions to the prohibition of GM crops in organic agriculture, allowing veterinary medicines, feed and food additives derived from GM crops, if there are no GM-free alternatives available. Absolute avoidance of GM crops is therefore not an imperative consideration for consumers of organic products in the EU (McEachern and Mcclean, 2002).

New plant breeding techniques (NPBTs) are most promising for organic farming. NBPTs, like cisgenic technology is being advocated as non-GM by some researchers (Lombardo and Zelasco, 2016; Ryffel, 2017). If this interpretation is accepted in IFOAM draft position paper “Position on Genetic Engineering and Genetically Modified Organisms,” IFOAM (2016) then it would, in principle, accept genetic transformation ‘technique’ as a potential tool for creation of ‘orgenic’ crops (Ryffel, 2012) – GM crops which are compatible with the standards of organic farming. Late blight resistant cisgenic potatoes, wilt-resistant bananas and golden bananas are examples of candidate orgenic crops that could be acceptable for organic farming (Paul et al., 2016; Dale et al., 2017; Gheysen and Custers, 2017). The opponents argue that unless the culture within the GM research arena changes, NBPTs will fail to meet the standards laid out by IFOAM in much the same way as existing biotechnologies today (Wickson et al., 2016). Ceccarelli (2014) argues in support of the idea of evolutionary-participatory plant breeding because it is a relatively inexpensive strategy to ‘adapt’ crops to both abiotic and biotic stresses and to organic agriculture, and directly generates varieties in farmers’ hands. There seems to be a middle path suggested by Dr. M. S. Swaminathan, who argues in favor of public sector coordinated trials (discussed below).

## Should an ‘Unknown’ Risk Warrant Over-Regulation, Especially in the Public Sector R & D?

Despite promising results, GM crops have not delivered their full promise, especially in the context of developing countries, due to the high cost of regulatory compliance (e.g., up to US$20 million to gain commercial certification of a single GM crop), and the ‘fear of the unknown,’ which may result from cultivating GM crops (Borlaug, 2000; Potrykus, 2001). This is not withstanding a report by the European Commission nearly a decade and half back (2001), assuring the safety of GM crops and food, based on research spanning 15 years, involving 81 projects with 400 scientists. The report concluded that GM plants have not shown any additional detectable risks to environment or human health, beyond the typical uncertainties of traditional plant breeding (reviewed in Husaini and Tuteja, 2013). Even the former founder of the Greenpeace movement, the major organized opponents of the GM technology, Dr. Patrick Moore, denounced Greenpeace as committing a “crime against humanity,” for opposing the use of GM crops, such as Golden rice^2^. Moreover, recently, over 100 Nobel Laureates urged Greenpeace and its supporters to abandon their campaign against GM crops in general, particularly Golden rice crops^3^.

1st World Food Prize Winner Dr. M. S. Swaminathan has advocated promotion of more public sector research in GM technology so that there is an increased inclusiveness in access to this technology. According to Dr. Swaminathan, “*private companies will obviously produce technologies where the small farmer will have to buy the seeds every year and where the findings are protected by intellectual property rights. There is very good expertise in our public sector institutions in the fields of molecular biology and genetic engineering and we should derive full benefit from them.”* Collectively, there is now sufficient scientific data and arguments that should prompt political determination to develop GM crops in the public sector; else excessively high costs of regulation would be a blessing in disguise for multinational agriculture companies to keep hold of the GM crop development. To some extent, it would perhaps address the issues of ‘cultural change’ (Wickson et al., 2016) as well as ‘evolutionary-participatory plant breeding’ (Ceccarelli, 2014).

## Transgenics for Organics: Is This the Way Forward?

Production of organic crops requires more land than GM crops, and according to Norman Borlaug, universal organic farming will only feed up to 4 billion people^4^. Overall, organic farming will require an increase in animal population to meet manure demands, which is self-defeating as sustaining a vast animal population will not only cause biodiversity loss by over-grazing, but also be a major contributor of greenhouse gasses. A meta-analysis of over 70 peer-reviewed studies revealed that organic products are not necessarily better for the environment. Organic cereals, milk, and meat generated more greenhouse gas emissions per product than their non-organic equivalents (Tuomisto et al., 2012). Can the above problems be addressed using GM crops to reduce the environmental footprint per unit of production without adversely affecting biodiversity? It was established in the meta-analysis by Tuomisto et al. (2012) that, per unit of product, organic produce generates higher nitrogen leaching, nitrous oxide emissions, ammonia emissions, eutrophication and acidification potential than conventional methods. Available solutions could include cultivation of GM crops in organic systems, as this would in fact be in tune with the ‘principles’ of organic farming, i.e., reducing environmental footprint and increasing sustainability. For example, the United States has set a trend by increasing GM crop production, valued at US$150 billion, creating a better environment as a result by saving 584 million kilograms active ingredient of pesticides in 2014 alone, in turn reducing CO_2_ emissions by 27 billion kilograms, the equivalent of taking 12 million cars off the road for 1 year^5^.

There is not simply one way of organic or conventional farming as there will always be compromises to be made; therefore, the debate needs to go beyond ‘organic vs. conventional.’ Nobel Laureate Norman Borlaug, the father of the Green Revolution, once commented that “*GMOs can play a very vital role in people’s lives. However, this must be accompanied by political goodwill because technology alone cannot survive without decisive support*” (Husaini and Tuteja, 2013). We cannot afford to prolong the wait of incorporating GM crops into organic farming methods endlessly just because of the so-called “precautionary principle”!

## Author Contributions

All authors listed have made a substantial, direct and intellectual contribution to the work, and approved it for publication.

## Conflict of Interest Statement

The authors declare that the research was conducted in the absence of any commercial or financial relationships that could be construed as a potential conflict of interest.

## Footnotes

1 http://www.greens.org/s-r/gga/fox.html

2 http://www.independent.co.uk/news/

3 https://www.washingtonpost.com/news/

4 https://www.technologyreview.com/s/409243/green-revolutionary/

5 https://www.isaaa.org/resources/publications/briefs/51/
